# The Causes of Irregularity in the Development of the Teeth

**Published:** 1875-09

**Authors:** N. W. Kingsley

**Affiliations:** New York.


AMERICAN JOURNAL

OF

### DENTAL SCIENCE.

**Vol. IX. THIRD SERIES—SEPTEMBER, 1875. No. 5.**

### ARTICLE I.

### *The Causes of Irregularity in the Development of the Teeth.*

[Concluded.]

BY N. W. KINGSLEY, D. D. S., M. D., NEW YORK.

I do not find in any of my reading that any author on the causes of malposition of the teeth has made this direct connection between the abnormality and disturbance of the nerve center during the formative and eruptive period; but I do find a large array of facts, confirmed by my own observations, which point in my own mind to this only conclusion, and although other observers of similar facts have attempted in many instances an explanation of what they saw, they have failed to refer them to any satisfactory *primary* cause.

In Mr. Mummery's paper, before referred to, in speaking of *diseases* of the teeth, he says, "It is to be feared that a large amount of dental disease is originated by overtaxing the active brain of children. According to the best authorities, the most rapid increase in the growth of the brain takes place before seven years of age; and it must be remembered that the crowns of all the permanent teeth, with the exception of the third molars, are simultaneously in the course of development with this great advance in the size of the brain. May we not, therefore, reasonably suppose that through the diminished vitality consequent upon this diversion of the formative energy from the teeth, by premature mental exertion, these organs necessarily become degenerated, and that this circumstance constitutes one great difference between the teeth of the intellectual and those of the uncultivated families of mankind?"

Dr. Nathan Allen, in his remarkable discourse delivered before the Massachusetts Medical Society, commenting upon the change going on in the human organism of the present day, says,—

"This charge indicates the existence of less muscle, more nerve; less physical vitality, more nervous energy; less power of endurance, but more mental activity.

"This same change is also indicated in the anatomy and physiology of the person. The framework of the body generally is not so large, is not so compact, nor so well proportioned; the countenance is paler, the features are more pointed and not so expressive of health, though more so of intelligence. The texture or quality of organization is more delicate and refined; the brain is becoming developed more and more relatively, and too frequently at the expense of the body; or, in other words, the nervous temperament, with all its advantages and disadvantages, is becoming too predominant for other parts of the body. As one of the consequences we have more diseases of the brain and nervous system, more sudden deaths from apoplexy, paralysis, and also from diseases of the heart. * * *

"The removal of so large a proportion of the population from the country and rural life to cities and large towns; the change of employment from farm-work,—from out-door exercise and the more laborious mechanical pursuits,—to lighter kinds of business, with increased exercise of the brain; add to this the greatly increased strife, excitement, and competition in every department of business and society, —all these changes must serve gradually to diminish muscular power and the general vitality of the system. No truth in vital statistics is better established than the fact that large cities and a dense population tend to diminish the physical energies of the body and shorten human life. * * *

"The simple reason is, as we conceive, that their style of living taxes the brain altogether too much; it develops a great predominance of the nervous temperament at the sacrifice of other parts of the body, which by inheritance is increased from generation to generation. The balance of structure and harmony of function in organization is radically changed, and carried to an intense development of nervous tissue, which in its very nature is unfavorable to the procreation of offspring. * * *

"But it is in the accumulated, the intensified effect produced by the law of inheritance, that the most striking and destructive results are to be witnessed."

My argument from this now almost universally recognized condition is this: during the formative and eruptive periods of the permanent teeth they are under the influence of an independent and peculiar vital (nervous) force; this innervation pushes on their development regardless of the more tardy growth of the osseous system; being implanted in a crowded position, in undeveloped maxillæ, they never have an opportunity to recover from it, and emerge in the same disordered arrangement in which the crowns were formed. The correlation and the connection would seem to be unmistakable. Under such circumstances it would not be expected that any or all organic changes in the nerve center would manifest the same results in detail; a disturbance of function would produce general results, the details of which might vary in every case.

That such a lesion or innervation could only operate upon the permanent teeth is easily seen, when it is remembered that to produce any marked effect upon the deciduous teeth, we should have to go back to intra-uterine life for the period of its influence, and before the child had an independent and sentient being.

This hypothesis does not find any seeming contradiction in our daily observation, for such a disturbance of nerve function might occur only for a limited period, and no other exhibition or evidence of it ever again appear.

In meditating some time since upon the subject, I came to the conclusion that, if my deductions were correct, I should find proof of it in an examination of individuals of sluggish or feeble intellect, and at that time I wrote this sentence:

"In an examination of idiots, we shall be likely to find capacious jaws and teeth not crowded."

If a precocious or stimulated brain in infancy urges on and crowds the dental organs in advance of the growth of the jaws, then a brain of low calibre, or power, will be likely to have associated with it a retarded dentition, but with abundance of room.

After this conclusion of my own, I came across a paper by Dr. Langdon Down, Physician to the Earlswood Asylum for Idiots, near London, and read before the Odontological Society of Great Britain.

Dr. Down's statement, founded upon his observations of nearly a thousand feeble minded patients, were so astounding, and so at variance with my theory, that the picture which I had painted seemed to vanish like a vision.

His most startling statement was embodied in these words:

"A marked character of the teeth of idiots is their irregularity as to position. They are often crowded,—so crowded as to present their sides instead of their anterior surfaces.

"They are often arranged on different planes. The canine teeth are frequently unduly prominent, and a marked sulcus is sometimes seen between the incisors and canines, with prominence of the incisors. * * *

"Of the most significant value, however, is the condition of the palate. I have made a very large number of careful measurements of the mouths of the congenitally feeble-minded and of intelligent persons of the same age, with the result of indicating, with some few exceptions, a markedly diminished width between the posterior bicuspids of the two sides." * * *

"One result, or rather one accompaniment, of this narrowing, is the inordinate vaulting of the palate. The palate assumes a roof-like form. The vaulting is not simply apparent from the approximation of the two sides; it is absolute, the line of junction between the palatal bones occupying a higher plane. Often there is an antero-posterior sulcus corresponding to the line of approximation of the two bones." * * *

So constant and universal did Dr. Down state that he found irregularities of the dental organs of one form or another, that he relied upon the evidence of the mouth as a valuable factor in making his diagnosis of the character of the idiocy. This essay, coming from so distinguished an authority, called forth an article "On the V-shaped Contracted Maxillæ," from the pen of Mr. Charles Tomes, who undertook to trace these phenomena back to their origin and explain them.

This contracted or V-shaped arch, I gathered from Dr· Down's paper, was the most inimical and the most pronounced of all deformities of the dental organs, and this statement in particular wast he most contradictory of my own hypothesis and of m*y* personal observations: for during a period of twenty-five years I had been noting and treating all forms of abnormalities, and the V-shaped or contracted arch was universally associated with a higher order of intelligence. On the very day of this writing, I examined the mouth of a lady in which there was seen contracted and V shaped arches in both upper and lower jaws of most decided character. The lady was one of unusually well-balanced mind and of a superior order of intelligence. No ordinary observer could possibly associate any feebleness of intellect with the abnormality in the person of this lady.

To gather evidence from a more extended field, and, if possible, to reconcile seeming inconsistencies, I undertook an investigation which has been continued with more or less interruption until the present time.

In entering upon an investigation of this kind, with a view of demonstrating a fact in science, it is of the utmost importance that there be no ambiguity of language or terms.

I have heretofore spoken of the normal type of the dental arch being a regular curved line.

I wish now to state that the absolutely, perfectly regular line is very rarely found in either ancient or modern skulls. To be more explicit, it will be found that nearly every case which would be pronounced regular by an expert on looking at it, will show variations when put to a mathematical test.

For instance, if a piece of soft wire be bent around the outside of the circle so as to touch every tooth, it will show places indicating that certain teeth are either within or without the line, and yet the deviations are so little as to be hardly observable by the ordinary critic.

Such deviations as this I do not class under the head of irregularities, because they are no more out of the limit of normality than are the varieties of feature in different members of the same family.

Furthermore, it is not certain that they are in any sense developmental in their origin, and if not they cannot be used as an argument in an investigation into development.

My own opinion is that, with the exception of inherited deformities, almost all cases of slight peculiarities of position result from the accommodation of articulation with the opposing jaw.

We remember their anomalous position before eruption; if they continued to grow in the same direction, great general disorder would be the result.

That they ever come perfectly into line, is due partly to the enlargement of the jaw encouraging it, and partly from articulation with the antagonizing teeth on occlusion. As it is only *inclined* surfaces that come in contact, we can readily see that a jaw which has the full measure of natural tendency to regularity, may thus show some deviations, as the permanency of the structure is attained.

A peculiar rotary movement in masticating, when teeth are erupting, may be sufficient to throw a bicuspid ever so slightly out of position, which malpostion may be considerably increased or it may be corrected, by contact with the opposite tooth on occlusion.

In the investigations I am about to describe, I used pieces of thick card-board cut about two and a half inches square, which were inserted in the mouth, the teeth closed, and with a pencil a line was drawn around the circle and close to the teeth. In such a diagram both the size of the arch and its form can be studied.

My first visit was to the inmates of the Asylum for Idiots upon Randall's Island, numbering about two hundred. The result was as follows:

I did not see a single pronounced case of V-shaped dental arch; I saw but very few cases of narrowed palatine arch; I saw but three or four of saddle-shaped palates, *i. e.* where the sides of the palate approximated in the bicuspid region.

I saw but little irregularity in the positions of the teeth; very few teeth that were out of line, whatever that line was; what little malposition there was, was generally confined to the six front teeth; of these six, the lateral incisors were more generally at fault, being inverted, everted or twisted. It was not a common thing to find the canines out of line.

Many of the malposed cases were those where the teeth were still erupting, and did not show such abnormal position that I would have felt justified in interfering if the patient had been brought to me for treatment. There was every reason to hope that, when fully developed, they would appear in good position. There was also many cases of retarded dentition.

There was no more irregularity, decay, loss of teeth, or neglect, than I have seen in the same number of youth picked up from the street.

The prevailing impression was, that I had seen an unusual number of well-developed jaws; they would average larger than the fully developed jaws of the average of my patients; and that they were above the average for density and probable durability.

The dental arch was generally a broad and regular curve; the variations from this, but within the range of normality, were a smaller circle, anterior to the bicuspids, and straighter lines behind them.

The lower jaw corresponded with the form of the upper in nearly every case, and therefore the articulation was almost always *good.*

There were three or four cases (the ones with saddle-shaped palates) where there was a narrowed or somewhat pinched condition of each side against the first molar, with which the lower jaw did not correspond, and the lower teeth articulated there outside the upper ones. There were no cases (save that of John Rawse) where there was any more deformity of contracted sides than is seen in the cases which I have treated, and which are described and illustrated in *Johnsston's Dental Miscellany* for the current year.

Associated with the last-named peculiarity, there were four or five where the superior incisors appeared tipped up, as in the so-called thumb-sucking cases, and which it is quite possible were caused by sucking habits; in some of them a vertical gap, of from one-quarter to three-eighths of an inch, between the incisors of both jaws when the teeth were closed, was observed.

There were several cases where the arch was well curved upon the right side, but the left side showed a variation from that curve and a depression of the line. There were more cases where the arch was depressed on only one side than upon both.

I did not see a single case in which there was any abnormality of size or shape of the jaws before the eruption of the permanent teeth.

I was informed that a very considerable number of these patients are of Hebrew extraction. The others are made up from nearly all civilized nationalities.

These conclusions differed so much from Dr. Down's observations, that I almost doubted my knowledge of what constituted normality and what constituted depravity.

To familiarize myself further with the subject, I went into a somewhat extended investigation of different classes, conditions, and races. In the youth of our public schools, above twelve years of age, I found ample facilities of one kind, and among the various nationalities and races represented in our metropolis, I carried the investigation further. For example: I hunted up every "*Heathen Chinee*" that I could find in New York (and there is a colony of about three hundred,) and took a diagram of his mouth. Recently while spending a number of weeks in Switzerland, I devoted some time to an investigation of Cretinism, and later I obtained authority from the Public Administration of Paris to visit the asylums and hospitals, and make an examination of idiots.

Assisted by Dr. John W. Crane, who rendered me most valuable aid, I made a personal examination of between three hundred and four hundred idiots, with a result in nowise dissimilar to that narrated somewhat in detail of the asylum on Randall's Island, and therefore unnecessary to recapitulate.

By a comparison of my observations of idiots with that of all ranks and conditions in life, as reprerented in our public schools, I found that, take the idiots as a class and compare them with the lower orders of society as found in this country, and there were no more irregularities in the one than in the other.

In both cases did I find that amply-developed jaws and teeth were the rule, and narrow, pinched, and V-shaped maxillæ and dental arches were the exception. And this was equally true of the idiots in France and of the Cretins in Switzerland, among whom I did not see a single case of narrowed arch or high vaulted palate.

From conversations with those in charge of the educa tion of the idiots, I was able to note the intellectual status of the patient and the corresponding condition of the dental organs. It was thus I discovered, that in those cases where there was a fair physical constitution and development, the intellect in a progressive state, and considerable hopes entertained of mental improvement, the jaws and teeth were in a normal condition. As the scale descended until we arrived at that melancholy condition of absolute idiocy, upon which all improvement was hopeless, I found jaws and teeth, and in a measure, the whole physical condition, degenerate. Thus did I see the extremes and the gradations beginning with a sluggish or feeble mind, in a fair organization with well developed jaws, descending in regular sequences through all the grades of imbecility to unconditional vacuity, associated with corresponding disorganization and degeneracy of the teeth, jaws, and whole physical system.

This experience had been so uniform on both sides of the Atlantic, that it was with feelings of hesitation as well as of desire that I accepted an invitation from Dr. Down to visit his asylum, near London, and see for myself the evidence upon which his convictions were based.

Together, we made a careful examination of every inmate of the institution, with a result not so widely different as I had supposed must exist.

There were to be sure, a larger percentage of irregularities of the teeth than I had before observed. About five per cent. might be said to be pronounced cases of narrowed or V-shaped arches, and another five to ten per cent. might be said to have more or less tendency in that direction, but of the more positive cases I did not see one so marked as I have seen and treated in private practice, and associated with full intellectual development.

Of the total number, I could not pronounce one-half, or fifty per cent., as showing an irregularity of dental development out of the range of normality, or that might not have arisen from accidental causes operating on the crowns of the teeth after eruption, and therefore only incidentally connected with idiocy, and in nowise correlated thereto.

In justice to Dr. Down, I must say that I found in him an enthusiastic co-operator, without any pet hobby to sustain against evidence, and only too glad to obtain aid in his investigation into the symptoms of idiocy from any source.

It was with the hope of obtaining aid that he went before the Odontological Society, with the expectation that the observations of experts in dentistry would throw some light upon the subject.

In this way only is to be accounted for the difference of opinion which existed between us upon many cases which seemed to him an unduly high vault to the palate, narrowed arch, or irregularity, but which, to my eye, came clearly within the limit of normality. Nevertheless, there was no mistaking the fact that there was a larger percentage of the kind of deformities which he had described, than I had found in any other collection of idiots. This fact was the less contradictory of my former experience, and the less puzzling, when I learned that nearly all the cases which I had seen were the offspring of noble families, or of the higher orders of society, which, in Great Britain, are more strongly marked than in any other portion of the civilized globe.

From one of Dr. Down's conclusions I must still dissent. In his essay, he says, "It was in my inquiry into the condition of the teeth and mouth especially, that I arrived at the conclusion that, in by far the larger number of instances I was able to indicate the period at which the depressed condition commenced, and to predicate in some degree the amount of improvement which physical, intellectual, and moral training might possibly effect.

"In children where idiocy is accidental, arising from causes operating after uterine life, there is but slight deviation from normal condition in the state of the mouth and teeth, while it is in those whose malady is congenital, especially where arising from causes operating at a very early period of embryonic life, that the deviation of the mouth and its appendages from a normal condition is most pronounced."

I cannot see any connection between these phenomena and causes acting especially "at a very early period of embryonic life;" when we consider that the deciduous teeth are well placed, and that it is only after they pass away that abnormality appears, also that it is only at the very latest period of embryonic life that even the germs of the permanent teeth are found, it is difficult to conceive the connection or correlation.

If the cause antedates the birth of the child, I should seek for its origin, not during the intra-uterine life, but as a like deformity existing in the progenitor, and associated with a *tendency* to idiocy,—developing into idiocy in the child, and carrying with it the physical deformity of the parent.

If there was always a disturbed condition of the deciduous teeth, we might pronounce on the congenital origin of the idiocy without hesitation, but when we find the deciduous teeth regular, and the permanent ones only irregular, we cannot find in this, evidence of nearly embryonic disturbance.

With the panorama before us of the development of both sets, we can only say with confidence that the abnormality of brain did not influence the position of the teeth until about or after the period of birth,—being the period covered by their growth.

Not long since, Mr. C. S. Tomes published an ingenious and somewhat elaborate theory, "On the Development

Origin of the V-shaped Contracted Maxilla," basing his arguments upon the investigations of Dr. Down.

Instead of accepting his hypothesis as justified by the premises, I am confirmed in the belief that wherever such abnormalities as he describes appear in unmistakable congenital idiots, they will be found peculiarities of transmission by inheritance,—like deformities existing in the ancestry.

Again, Dr. Down says, "An appeal to the condition of the mouth is an important aid in determining whether the lesion on which the mental weakness depends is of intra-uterine or post-uterine origin. In the event of the mouth being abnormal, it indicates a congenital origin; while if the mouth be well formed, and the teeth in a healthy condition, it would lead to the opinion that the calamity had occurred subsequently to embryonic life."

This opinion of Dr. Down is exactly the opposite of my own. There are so many cases where the proof is incontestable that the idiocy is of congenital origin, and where the dental development is perfect, that if I were to draw any proof from the facts, I should say that the regularity of the dental organs showed conclusively that there had been no lesion or disturbance of the brain (in the true sense) after birth, and that the teeth grew regularly in accordance with physical laws under a low order of intellect.

These remarks, of course, do not apply to epileptics, nor to imbeciles who were bright in infancy, and from any cause, afterward, degenerated.

Let it be borne in mind that no irregularity in the position of the dental organs is any evidence of idiocy in the individual. The cases are so common where such deformities are found associated with the highest order of intelligence, that if I were to draw any inference from that condition in the abstract, I should say that it was more likely to indicate a precocity of mental development in the child, and very possibly a more brilliant intellect in the adult. At all events, it does conclusively prove a disturbed cerebral condition at some period in the child's history; or, if it is the result of hereditary taint, shows such a condition in the progenitors, which has originated from like causes, and, unless checked, will become intensified by transmission under similar surrounding conditions; and the future history of that family will be mental degeneracy.

An inherited taint, disturbance, lesion, predisposition, or tendency to idiocy, of which these irregularities are only a symptom or proof, may show itself in a precocious mental development in one instance, and be the precursor of insanity in the same individual, or appear as idiocy in posterity.

A resume of my experience and observations shows the following results. In private practice I have seen a very large aggregate of dental deformity, and in most instances associated with an intellectual capacity above the average of mankind.

These abnormalities have not been confined to any prevailing type, but have included nearly every possible variety of irregular development. In the public schools, among the middle and lower classes of society, I have found but a small percentage of pronounced irregularity. Such as I have found was in nearly every case among the brightest children of the schools.

Among the children of good physique and fair mental capacity, the development was on the whole regular and normal; a noticeable fact being that the jaws were generally capacious and ample for the regularity of the teeth. In an occasional instance, there might be found a jaw of undue capacity and with separated and straggled teeth. One instance of this kind was particularly marked, and on making inquiry of the principal as to the status of the scholar, I was told that she was the dullest one in the school of over three hundred, and, although of an age to justify an entrance into the highest class, she could not rise above the lowest.

In the examination of idiots I have endeavored to separate those where the mental defect dated from birth, from those who in infancy showed the average mental capacity, and who from accident or disease afterward degenerated into imbeciles.

No more reliance is to be placed upon an examination of the jaws and teeth of the latter class, in a question of development, than upon an examination of the inmates of an insane asylum.

Confining myself to congenital idiocy I found only a small percentage of pronounced irregularity in form of the jaws or arrangement of the teeth, and that generally associated with the lowest type of idiocy and the kind of which mental improvement was the most hopeless. Associated with deformities of the jaws were usually other abnormalities of physique showing a general constitutional disturbance.

Among the Cretins, capacious jaws were universal. Only among idiots drawn from the higher classes in Great Britain were there found exceptions.

My collection of evidence on this subject closes with another extract from Mr. Mummery's paper: "The Australian races exhibit in the configuration of the skull the lowest type of the human family. The cranium in these people is very narrow, the forehead low and receding, the teeth—especially the canines—project in a striking manner, and other irregularities were frequent.

"The physical character and habits of the Tasmanian race (now nearly extinct) so closely resemble the Australian that I need only observe that in all points relating to disease, irregularity, etc., the defects of the Australian are exaggerated in the Tasmanian."

The deductions which I make from this array of facts, made up from all ranks and conditions of life, including races, nations, and classes,—ancient and modern,—from the highest order of intelligence down to the idiotic, are as follows:

A perfect dental development is the result of well-balanced physical and nervous systems, without hereditary taint.

The causes of irregularities I classify as developmental and accidental: the developmental operating prior to the eruption of the teeth, and the accidental subsequently.

Abnormalities of development having their origin in the same individual are due to a disturbance of the trigeminal nerve during the period in which the crowns of the permanent teeth are forming and arranging themselves in the jaw prior to eruption; or, when arising from causes antedating the life of the individual, are traceable to an inherited tendency, which tendency had its origin in a like disturbance in one of the progenitors, and was subsequently transmitted or are the result of mixing different and distinctly-marked types of jaws and teeth by the progenitors.

In our view we do not call a feeble mind, a sluggish brain, or a dull intellect, a nerve lesion or a brain disturbance. For it is abundantly proven that when this condition is associated with an average physique, the development of the dental organs is tardy, but in regular order.

We have before us, then, both the solution of the problem and the evidence of most alarming symptoms in the physical and mental condition of the inhabitants of the centers of civilization.

There can be no question that the Creator intended there should be perfect harmony in the development of physical and nervous systems, and that where such harmony exists we come nearest to the standard of a perfect organization. This harmony of organization or true balance of the two systems demands that in the earlier years of life the brain and the nervous system be held in abeyance to the physical.

The healthier mental organization is of slower growth. If, therefore, we find that a certain mode of life destroys this harmony, breaks up this balance, there will follow necessarily deterioration and destruction of the race; and this is based on well-recognized physiological law; if the brain and the nervous system are in an undue state of activity, the drain upon the sources of nutrition will be at the expense of the physical system.

No force operating on the brain can interrupt or alter the type, or inherited model of the dental arch, after the first decade of life. All brain disturbances occurring during that period showing mental aberration, we should class under the general head of idiocy—imbecility. After that period, such manifestations come more properly under the head of lunacy,—insanity,—which might degenerate into imbecility or idiocy.

Consequently, neither lunacy nor insanity, in the ordinary acceptation of the terms, can have any direct bearing upon the development of the dental organs; but such a condition would be most potent of evil if transmitted to offspring.

*I do not hesitate to place it upon record that the next generation will see more of abnormality in dental development, and an increase of nervous and cerebral diseases, and that the two are correlated and spring from the same cause.*

It is too late to stop it in those who have passed infancy, but it is not too late to modify and partially remedy the evil in those now being born, and those who may be begotten hereafter.

To fathers and mothers surrounded by luxury and flattered with the precocity of their infants, which they are stimulating to the last degree, I say, You are the enemies of your race; you are sowing the seeds of nervous, mental, and physical disorders, from which the harvest will be fearful, and the end death to your family and your name. Do not, under peril, encourage this brilliancy of your child, which is now so charming; let the mind stagnate rather.

For the first seven years of life give concern *only* to his morals and to his physique. Nourish him as you would nourish an animal from which you desired the finest development, stimulating *only* his moral nature, and his intellect will take care of itself. Thus, if we have no hereditary taint, you will have laid the foundation of a splendid specimen of his race.

